# Bio-Source of di-n-butyl phthalate production by filamentous fungi

**DOI:** 10.1038/srep19791

**Published:** 2016-02-09

**Authors:** Congkui Tian, Jinren Ni, Fang Chang, Sitong Liu, Nan Xu, Weiling Sun, Yuan Xie, Yongzhao Guo, Yanrong Ma, Zhenxing Yang, Chenyuan Dang, Yuefei Huang, Zhexian Tian, Yiping Wang

**Affiliations:** 1 College of Environmental Science and Engineering, Peking University; Key Laboratory of Water and Sediment Sciences, Ministry of Education, Beijing 100871, China; 2 School of Environment and Energy, Peking University Shenzhen Graduate School, Shenzhen 518055, China; 3 State Key Laboratory of Hydroscience and Engineering, Tsinghua University, Beijing 100084, China; 4 College of Life Sciences, Peking University, Beijing 100871, China

## Abstract

Although DBP (di-n-butyl phthalate) is commonly encountered as an artificially-synthesized plasticizer with potential to impair fertility, we confirm that it can also be biosynthesized as microbial secondary metabolites from naturally occurring filamentous fungi strains cultured either in an artificial medium or natural water. Using the excreted crude enzyme from the fungi for catalyzing a variety of substrates, we found that the fungal generation of DBP was largely through shikimic acid pathway, which was assembled by phthalic acid with butyl alcohol through esterification. The DBP production ability of the fungi was primarily influenced by fungal spore density and incubation temperature. This study indicates an important alternative natural waterborne source of DBP in addition to artificial synthesis, which implied fungal contribution must be highlighted for future source control and risk management of DBP.

Phthalate acid esters (PAEs) are pollutants that are often continuously released to the environment in effluent from industrial processes^1^. Of PAEs, di-n-butyl phthalate (DBP) is particularly widespread and readily detectable in many water bodies throughout the world. In many cases, the DBP content has been found to exceed substantially the safe limits defined by national and international standards^2^. In industry, DBP is utilized as a plasticizer, as a solvent, and often added to inks or adhesives. Its primary applications are in the manufacture of plastics, textiles, paints, packaging of pharmaceutical products, toys, etc^3^. It is well established that exposure to DBP carries potential health risks to humans and animals, including endocrine system disruption, inhibition of enzyme activities in testes, and reproductive tract malformation and weight reduction^4^. Long-term exposure to DBP has been found to cause prostate hyperplasia and trigger an inflammatory response in mice^5^. DBP has an adverse effect on human and animal brain development by altering the function of the thyroid system, and it can reduce the detoxification activity of superoxide dismutase in organisms, leading to very adverse effects^6^. In recent years, biological monitoring studies have detected DBP in human body fluids (e.g. urine, serum, breast milk, and saliva). Concern about DBP has led to restrictions being imposed on its use in the manufacture of toys and prohibition of its use in cosmetics^7^,^8^.

Although DBP has been isolated from plants and microbes, it is unclear if DBP could be continuously excreted to supernatant. Previous studies have reported on the presence of DBP in different plants, such as *Salicornia herbacea*, *Ipomoea carnea stem*, and *Callianthemum taipaicum*^9^,^10^,^11^. However, it is difficult to judge whether the DBP originates from secondary metabolites of these plants or from the environment by absorption and accumulation given that DBP already exists in air and surface waters^12^. Natural product researchers have obtained DBP from purple calf liver bacteria (*Boletinus*), endophytic bacteria in sponges (*Zygomycale*), and mangrove endophytic fungi *(Penicillium*), but they did not further investigate whether DBP was produced by the microorganism or from the contaminated environment^13^,^14^.

In order to understand whether fungal metabolism could be one of the sources for DBP pollution in water, we cultured different species of filamentous fungi in both an artificial synthetic medium and natural water, and then analyzed the content of DBP in ethyl acetate (EtOAc) extracts acquired from all the fermentation broths of three representative fungi. To provide a better understanding of the biosynthesis of DBP isolated from fungi and other microbes, we also examined the activity of crude enzyme produced by the three species of filamentous fungi in a cell-free system and hence speculate on the biosynthetic pathway of DBP. Surprisingly, the results show that such fungi are capable of producing relatively large quantities of DBP. This study is of particular importance to DBP source identification and control in natural environments.

## Results

### Identification of DBP excreted from fungi

The three naturally occurring filamentous fungi strains *Penicillium lanosum* PTN121, *Trichoderma asperellum* PTN7 and *Aspergillus niger* PTN42 were cultivated respectively for 1–16 days. After ultrasonication, the secondary metabolites were extracted from culture broth and analyzed by HPLC (High Performance Liquid Chromatography). The peak at 52.1 min corresponding to the EtOAc extracts of the three species of fungi was higher compared to other peaks during the first 7 days (see Supplementary Fig.S1 online). The substance corresponding to the peak was isolated and identified as DBP. The *pseudo*-molecular ion peak at 279 [M + H]^+^ m/z in ESI-MS (Electrospray ionization mass spectrometry), corresponded to di-n-butyl phthalate based on its ^1^H and ^13^C NMR characteristics (1H NMR (400 MHz,CDCl_3_) δ:7.71 (2 H, m), 7.52 (2 H, m), 4.30 (2 H, t, J = 6.7 Hz), 1.71 (4 H, m), 1.44 (4 H, m, H-3), 0.96 (6 H, t, J = 7.4 Hz); 13 C NMR (100 MHz, CDCl_3_) δ: 167.8, 132.4, 131.0, 128.9, 65.7, 30.7, 19.3, 13.8)(see Supplementary Fig.S2 online). Satisfactory linearity was obtained between the HPLC response (peak area on chromatograms) and the amount of injected DBP standard. The regression line for DBP was obtained as *y* = 2490*x* (*r*^2^ = 0.9980) (2 mg/mL < *x* < 10 mg/mL) from the (*x*, *y*) plot, where *x* represented the concentration of the standard in milligrams per milliliter, and *y* represented the peak area. While the regression line for DBP was obtained as *y* = 2.546*x* (*r*^2^ = 0.9980) (20 μg/mL < *x* < 100 μg/mL) from the (*x*, *y*) plot, where *x* represented the concentration of the standard in microgrammes per milliliter, and *y* represented the peak area.

### Quantification of DBP production

**Figure 1:**
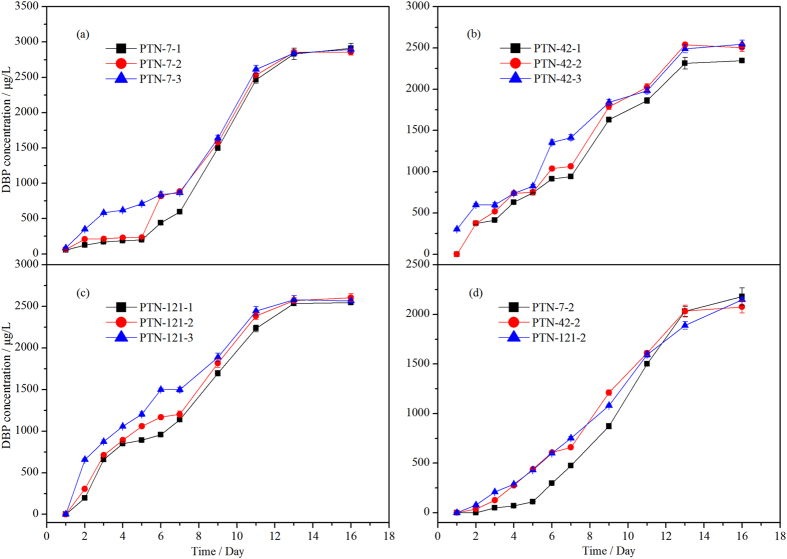
Temporal evolution of DBP production by three fungi inoculated in artificial media at 28 °C: (**a–c**) show the concentration of total produced DBP during the culture process inoculated with *T. asperellum* PTN7, *A. niger* PTN42 and *P. lanosum* PTN121, respectively. The labels 1, 2 and 3 refer to spore suspensions of 20 μL, 200 μL, and 2000 μL; (**d**) shows the concentration change of DBP in the culture medium without fungal mycelium during the culture process inoculated with *T. asperellum* PTN7, *A. niger* PTN42 and *P. lanosum* PTN121 at 200 μL spore suspension (See control in Supplementary Fig. S3, control 1).

**Figure 2:**
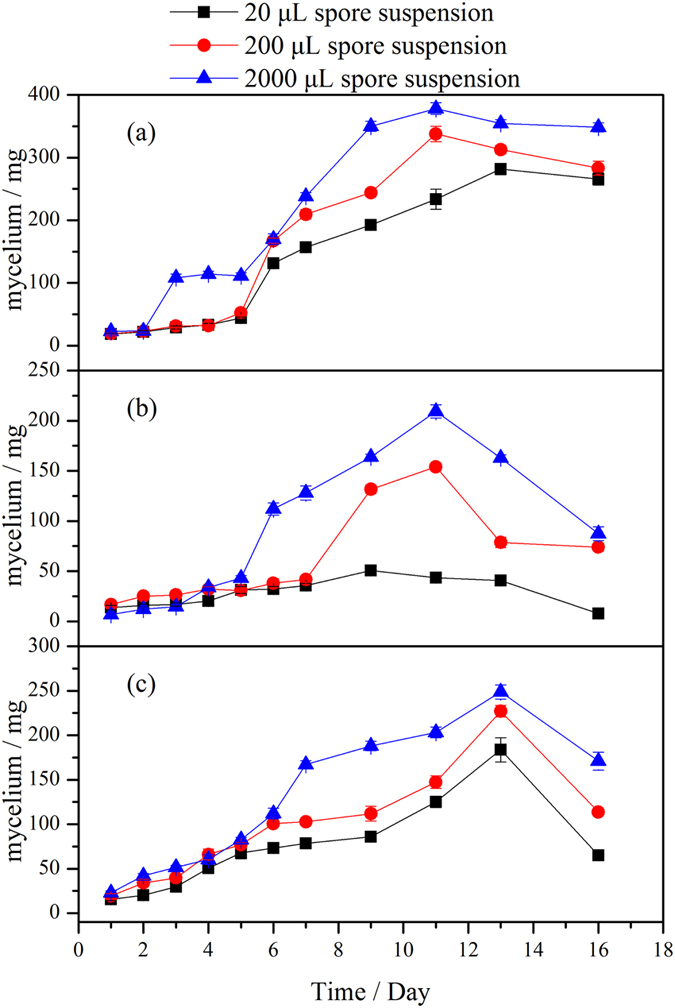
Dry weight of mycelium results for different spore densities by three fungi inoculated in artificial media at 28 °C from 20 mL fermentation broth.

EtOAc extracts of fermentation broth for the three strains at different spore densities were analyzed by HPLC (see Supplementary Fig.S1 online, and Supplementary Table S1 lists the spore numbers of the three fungi inoculated in the liquid medium). The peaks at 52.1 min suggest that all three strains were able to generate DBP, among which *T. asperellum* PTN7 at three spore concentrations and *A. niger* PTN42 at high spore concentrations could produce DBP after the first day’s fermentation (Supplementary Fig.S3 lists the controls). Supplementary Table S2 lists the key results for each strain obtained using regression analysis. Figure 1(a–c) shows a positive correlation between DBP production and spore density of the three fungi inoculated into each of 500 mL flasks with 200 mL of liquid medium; in other words, the larger the spore density, the greater the production of DBP. As the fermentation time increases, the effect of spore density on the production of DBP by the three fungi progressively diminishes. Without loss of generality, we analyzed the extracellular DBP in the culture medium which was inoculated with 200 μL spore suspensions (see Supplementary Fig. S4 online). Fig. 1 (d) shows DBP concentration in the culture medium (without fungal mycelium) was increased with increasing culture time. More importantly, increasing proportion of the extracellular DBP in the medium over the total produced DBP (Fig. 1(a–c)) was also observed, which reached 76%, 83% and 81% for *T. asperellum* PTN7, *A. niger* PTN42 and *P. lanosum* PTN121 respectively after 16 days’ cultivation. Supplementary Table S3 indicates dry weight of mycelium results obtained at different culture times for the three fungi. Figure 2 indicates typical trends that the dry weight of mycelia changed with culture time of the three fungi. As the culture time prolonged, dry weight of mycelia increased to a certain peak and then began to decline, this is fully consistent with the growth regularity of microbes.

HPLC analysis on quantification of DBP production by the three fungi was also made after fermentation in the liquid culture at different DBP background concentrations. The concentrations of DBP in water ranged between 0~100 μg/L^12^,^15^,^16^,^17^,^18^, and thus the DBP background concentrations herein were set to 0, 25, 50, 75, and 100 μg/L, respectively. The results presented in Supplementary Fig. S5 and Table 1 indicated that there was no difference in DBP production for the cases considered. In general, the natural water temperature is about 28 °C in summer and 15 °C in winter at the sampling sites in the Yangtze River. The experiments confirm that the fungus can produce DBP at 28 °C at different fungal spore density and background DBP concentration. Further tests demonstrate that the fungi would also produce DBP at 15 °C (Supplementary Fig.S6). Table 2 shows all three fungi tend to produce higher concentrations of DBP at 15 °C than those at 28 °C.

### Quantification of DBP generated from fungus in natural water

In order to determine whether such fungi can still produce DBP in natural water, three water samples with different physicochemical conditions were collected from the upper (Chongqing, W-CH), middle (Wuhan, W-W) and downstream (Zhenjiang, W-ZH) reaches of the Yangtze River (Table 3). The Supplementary Fig.S7 shows the HPLC quantification of DBP production by *P. lanosum* PTN121*, T. asperellum* PTN7 and *A. niger* PTN42, after fermentation in W-CH, W-W and W-ZH samples. The DBP concentration in the natural water sample, W-CH, was detected as 17.1 ± 0.8 μg/L, whereas the concentrations in fermented cultures of *P. lanosum* PTN121, *T. asperellum* PTN7 and *A. niger* PTN42 increased to 30.4 ± 0.8, 42.6 ± 1.2, and 21.2 ± 0.6 μg/L, respectively. In other words, the DBP content of the natural water sample W-CH increased by 24~149% owing to the presence of the different fungal strains.

Table 4 lists the DBP production by the three fungi in the second natural water sample (W-W), whose background DBP concentration was 11.4 ± 1.2 μg/L in the absence of the filamentous fungi. However, a 62~205% increase of DBP content occurred with addition of the fungi. Similarly, the content of DBP in the third water sample (W-ZH) increased by 49~176%.

### Enzyme-mediated reaction

**Figure 3:**
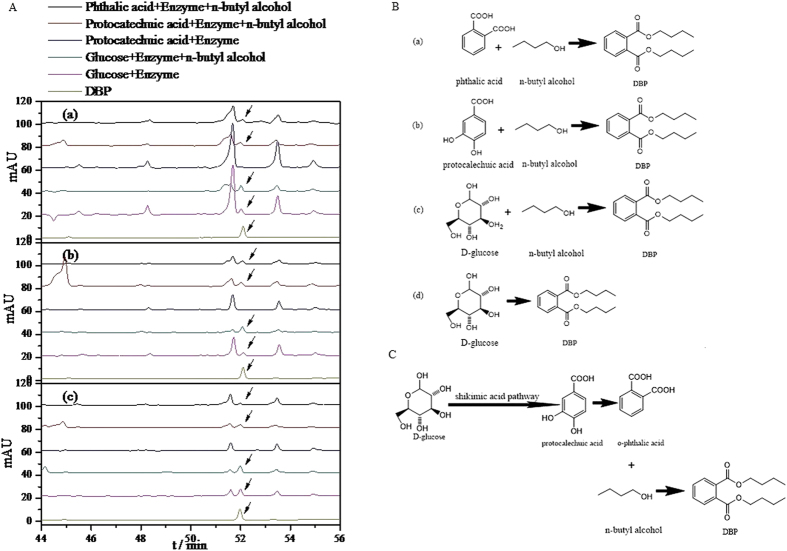
(**A**) HPLC time series of D-glucose, D-glucose and n-butyl alcohol, phthalic acid and n-butyl alcohol, protocatechuic acid and n-butyl alcohol for cases when the substrates were catalyzed by crude enzyme from the three different species of filamentous fungi: (a) *T.asperellum* PTN7; (b) *A.niger* PTN42; and (c) *P.lanosum* PTN121. Black arrows indicate the corresponding HPLC peaks of DBP (See controls in Supplementary Fig. S3, controls 2–7). **(B)** Biosynthesis of DBP catalyzed by crude enzyme from the three different species of filamentous fungi with different substrates: (a) phthalic acid and n-butyl alcohol; (b) protocatechuic acid and n-butyl alcohol; (c) D-glucose and n-butyl alcohol; and (d) D-glucose. **(C**) DBP biosynthesis of the filamentous fungi, *T.asperellum* PTN7, *A.niger* PTN42, and *P.lanosum* PTN121.

The degradation pathway of DBP as a kind of environmental hormone has been studied in great detail^19^,^20^. Nevertheless, the biosynthetic pathway of DBP was rarely studied. Based on the chemical structure of DBP (Fig. 3C), it is initially speculated that the shikimic acid pathway might be responsible for the gradual assembly of phthalic acid with butyl alcohol to eventually generate DBP. To test this hypothesis, D-glucose, D-glucose + n-butyl alcohol, protocatechuic acid, protocatechuic acid + n-butyl alcohol, and phthalic acid + n-butyl alcohol as the substrates with crude enzyme from the three species of filamentous fungi dissolved in phosphate buffer were incubated at 28 °C for 48 h, respectively. Figure 3A shows the results obtained from HPLC analysis of the EtOAc extracts. The peak at 52.1 min reveal that DBP production could be observed in D-glucose, D-glucose + n-butyl alcohol, protocatechuic acid + n-butyl alcohol, and phthalic acid + n-butyl alcohol catalyzed by crude enzyme isolated from the fermentation broths of fungi *P. lanosum* PTN121, *T. asperellum* PTN7 and *A. niger* PTN42, respectively. Figure 3B illustrates the chemical reactions that constitute the biosynthesis of DBP using the different substrates. It should be noted that DBP was not produced by the crude enzyme catalyzed reaction of any of the three fungi when using protocatechuic acid as the sole substrate. Although protocatechuic acid is an intermediate product obtained during the formation of DBP, no effect could be observed either with or without n-butyl alcohol added to the enzyme-catalyzed reaction, which converts D-glucose into DBP. Based on the structural characteristics of DBP, we surmised that n-butyl alcohol was also generated in the catalyzed reaction with D-glucose as substrate. This is a reasonable hypothesis given that many microbes produce n-butyl alcohol^21^,^22^,^23^. Although n-butyl alcohol can be produced by enzymes, experiments demonstrated that protocatechuic acid alone could not produce n-butyl alcohol, and thereby impossible to result in DBP. This is also partially supported by Curran^24^. It proved that the shikimic acid pathway was involved in biosynthesis of DBP, and the biosynthetic pathway of DBP with D- glucose as a substrate was hence speculated (Fig. 3C).

## Discussion

**T**he HPLC analysis confirmed that the three genera of fungal strains, *P. lanosum* PTN121, *T. asperellum* PTN7 and *A. niger* PTN42, can produce DBP at given fungal spore density (Supplementary Fig. S2), culture medium without fungal mycelium (Supplementary Fig. S4), background DBP concentration (Supplementary Fig. S5) and incubation temperature (Supplementary Fig.S6). Figure 1 shows that the DBP content and fungal spore density exhibited significantly positive correlation when fermented for about 10 days. This may be related to the fungal growth cycle reaching a certain stage at which cell autolysis commences and nutrients change in the culture medium. The increasing trend of DBP metabolites in the medium as shown in Fig. 1(d) as well as its high proportion to total produced DBP (over 75% after 16 days) implied the considerable contribution of DBP excreted out of the cells to water pollution. For all three fungi, the detected DBP concentrations were more than 25 times larger than the background values, demonstrating the large capacity of the fungi for DBP production. Remarkably, the concentrations of DBP produced by the three fungi cultured at 15 °C were higher than those at 28 °C.

Filamentous fungi are native inhabitants of water that are present almost everywhere, even in sulfidic spring waters^25^, saline marine waters^26^, and volcanic lakes^27^. In particular, the genera of *Penicillium*, *Aspergillus* and *Fusarium* are abundant fungi in water^28^. On the other hand, although occurrence, degradation, ecological damage and toxicity of DBP have been subjects of extensive research, little is known about the source of DBP pollution from microbial metabolites. The conventional view is that DBP is continuously released to the environment via effluent from industrial processes. However, limited papers reported that algae might produce DBP and possibly contaminate water^23^,^29^,^30^. In natural water containing lower carbon and nitrogen, fungi usually grow slowly. Our study provides unequivocal evidence that the three strains of fungi, *P. lanosum* PTN121, *T. asperellum* PTN7 and *A. niger* PTN42, are able to produce DBP in natural water sampled from the Yangtze River, which reminds us of the important contribution of the fungi for DBP generation in natural water environment. A symbiotic relationship is well known to exist widely between algae and microbes^31^ and so the true origin of DBP is worthy of further consideration. The key issue becomes if DBP is introduced during processing or really excreted by fungi. Our systematic investigation on three of the filamentous fungi which can generate DBP as cultured either in artificial medium or in natural water confirmed the metabolic ability for biosynthesis of DBP. To avoid cross-contamination by DBP, a glass container was used instead of a plastic one. All organic solvents (methanol, acetone, EtOAc etc.) used in this study were redistilled under reduced pressure. Moreover, crude enzyme produced by the filamentous fungi for catalysis of the relevant substrates in the cell-free system revealed the general biosynthetic pathway of DBP way as an alternative natural waterborne source of DBP pollutants in addition to artificial synthesis.

The DBP concentration in the upper (Chongqing, W-CH), middle (Wuhan, W-W) and downstream (Zhenjiang, W-ZH) reaches of the Yangtze River were detected as 17.1 ± 0.8 μg/L, 11.4 ± 1.18 μg/L and 12.4 ± 1.18 μg/L, respectively (Table 4), comparable to the maximum DBP concentration of 35.7 μg/L as reported in the literature^12^. Considering the species in the genera of *Penicillium* (relative frequency 16.8%), *Aspergillus* (7.6%) and *Trichoderma* (5.8%) respectively along the Yangtze River^32^, the DBP concentration of 7.6~9.9 μg/L excreted by the three fungi could be approximately estimated with the sum of the product, by multiplying the proportions of the three species (*P. lanosum* PTN121, *T. asperellum* PTN7 and *A*. *niger* PTN42) in the river and their corresponding DBP concentrations (30.4~34.8 μg/L, 18.5~42.6 μg/L and 18.9~21.2 μg/L, Table 4) in fermented cultures, respectively. Comparing the DBP excreted solely by the fungi and the maximal DBP monitored in the Yangtze River at similar conditions, the fungal contribution of DBP biosynthesis would be at least 21~28% in this third largest river in the world.

## Methods

### Chemicals and filamentous fungal strains

Authentic compound DBP was purchased from Dr. Ehrenstorfer GmbH (Germany). All of other organic chemicals used in this study were of guaranteed grade from Tainjin Guanfu Chemical Company (China). All organic solvents (methanol, acetone, EtOAc etc.) used were redistilled under reduced pressure. Fungal strains *P.lanosum* PTN121 (GenBank Accession No. KM594321), *T.asperellum* PTN7 (GenBank Accession No. KF589302) and *A.niger* PTN42 (GenBank Accession No. KF589309) were obtained by *a priori* separation. The strains were then cultured in potato dextrose agar (PDA) culture medium, and the test tube slant was stored in a refrigerator at 4 °C.

### Identification of DBP and DBP standard

All EtOAc extracts from the fermentation of three fungi, *P.lanosum* PTN121, *T.asperellum* PTN7 and *A.niger* PTN42 were subjected to HPLC analysis to detect the secondary metabolites produced by the fungi. HPLC profiles at 290 nm revealed that all the extracts produced a distinct peak with the same retention time of 52.1 min. The ^1^H and ^13^C NMR of the peak were recorded at 400 and 100 MHz, respectively, using a Bruker spectrometer. Serially-diluted stock solutions of DBP were prepared in methanol and 35 μL of each solution was injected into the HPLC column to create the standard curve from which the correlation was determined between peak area and amount of injected DBP standard^33^.

### Cultivation in artificial medium

Spore suspensions of the three fungi (2000 μL, 200 μL and 20 μL), *P. lanosum* PTN121, *T. asperellum* PTN7 and *A. niger* PTN42 were inoculated into a 500 mL cone-shaped flask with 200 mL of liquid medium (20 g glucose with finely-diced 200 g potatoes boiled in 500 mL of water until thoroughly cooked, and water added to filtrate to 1000 mL before sterilization, adjusted to pH 6.0 prior to sterilization), respectively, and fermented at 28 °C for 16 days on a rotary shaker at 150 rpm. Analysis was performed on 20 mL samples after 1 d, 2 d, 3 d, 4 d, 5 d, 6 d, 7 d, 9 d, 11 d, 13 d and 16 d of fermentation. Once fermentation was completed, a 20 mL sample of the broth was extracted using 30 mL acetone by ultrasonication for 1.5 h. The resulting aqueous acetone solution was then concentrated under reduced pressure to remove acetone, and the remaining water layer extracted three times using equal volumes of EtOAc to obtain the samples for DBP analysis by HPLC. For the broth which was added 200 μL spore suspensions, 20 mL sample was filtered after cultivation. The filtrate was then extracted by EtOAc for the analysis of DBP concentration in the culture broth. At the same time, 20 mL fermentation broth was oven-dried at 110 °C for 12 h after filtering with filter paper to obtain the dry weight of mycelium. 0, 5, 10, 15 and 20 μg of authentic DBP was added to 5 sterilized 500 mL cone-shaped flasks each containing 200 mL of liquid medium. *P. lanosum* PTN121, *T. asperellum* PTN7 and *A. niger* PTN42 strains were then fermented in the medium (which corresponded to DBP concentrations of 0, 25, 50, 75 and 100 μg/L, respectively), using the procedure as described above. Next, spore suspensions (200 μL) of *P. lanosum* PTN121, *T. asperellum* PTN7 and *A. niger* PTN42 were fermented at 15 °C for 30 days on a rotary shaker at 150 rpm, using the same cone-shaped flask as above with the same medium.

### Cultivation in natural water

Natural water samples were collected from the upper reach (Chongqing, China, W-CH), middle reach (Wuhan, Hubei Province, China, W-W) and downstream reach (Zhenjiang, Jiangsu Province, China, W-ZH) of the Yangtze River in March. The natural water was sterilized before the three strains, *P. lanosum* PTN121, *T. asperellum* PTN7 and *A. niger* PTN42 were fermented, using the procedure described in Section Cultivation of artificial medium.

### HPLC analysis

HPLC analysis was performed using a YMC-Pack Pro C18 analytical column (5 μm, 80 Å, 4.6 mm × 250 mm) in an Agilent 1200 HPLC system. 10 μl of each sample was placed in MeOH solution (100 mg/mL), injected into the column, and eluted with MeOH–H_2_O of linearly increasing concentration (from 20% MeOH at the initial time *t* = 0 min to 100% MeOH at the end time *t* = 60 min) in the mobile phase (flow rate, 1 mL/min). Diode array detector (DAD) readings were acquired and then processed by Empower DAD software (Agilent Corporation, USA) to obtain the target HPLC data.

### Crude enzyme preparation

The strains were inoculated individually onto fresh PDA plates and incubated for 4~8 days. Then fresh spores were inoculated into 500-mL Erlenmeyer flasks containing 200 mL fermentation medium. The medium consisted of 20 g glucose, 200 g boiled finely-diced potatoes, and distilled water to the final volume of 1 L. The cultures were incubated on a rotary shaker (150 rpm) at 28 °C for 4 days. Solid (NH_4_)_2_SO_4_ was added to the fermentation broth (after filtration) until the (NH_4_)_2_SO_4_ saturation reached 60%. The solution was mixed thoroughly for 5 hours, allowed to settle, and then centrifuged (12000 rpm at 4 °C) for 10 minutes. The resulting precipitate was dissolved in His-HCl buffer (0.02 mol/L, pH 6.5) and dialyzed overnight to obtain crude enzyme.

### Bio-transformation

For the analysis of crude enzyme activities, 10 mL of crude enzyme dissolved in phosphate buffer was incubated at 28 °C for 48 h with substrates comprising 10 mM glucose, 10 mM glucose and 10 mM n-butyl alcohol, 5 mM phthalic acid and 10 mM n-butyl alcohol, 5 mM protocatechuic acid and 10 mM n-butyl alcohol. The phosphate buffer was used as control. After extraction using EtOAc, the upper phase was collected by centrifugation and analyzed by HPLC.

## Additional Information

**How to cite this article**: Tian, C. *et al.* Bio-Source of di-n-butyl phthalate production by filamentous fungi. *Sci. Rep.* **6**, 19791; doi: 10.1038/srep19791 (2016).

## Supplementary Material

Supplementary Information

## Figures and Tables

**Table 1:** HPLC quantification of DBP production by three fungi at different background DBP concentration in artificial medium (times of repetition = 3).

Fungal strains	Background DBP concentration (μg/L)				
	0	25	50	75	100
PTN7	3031 ± 42	2958 ± 45	3003 ± 21	3055 ± 35	2922 ± 34
PTN42	2642 ± 19	2595 ± 31	2646 ± 56	2605 ± 36	2557 ± 41
PTN121	2850 ± 42	2796 ± 32	2822 ± 34	2893 ± 93	2768 ± 51

**Table 2:** Concentration (μg/L) of DBP by the three fungi cultured in artificial medium at 15 °C and 28 °C(repetition times = 3).

Strains	First test(μg/L)	Duplicated (μg/L)	Triplicated (μg/L)	Average (μg/L)
PTN7(15 °C)	5259	5276	5154	5230 ± 80
PTN42(15 °C)	14663	14677	15103	14810 ± 190
PTN121(15 °C)	5876	5936	5934	5920 ± 60
PTN7(28 °C)	2821	2847	2903	2857 ± 42
PTN42(28 °C)	2447	2545	2521	2504 ± 51
PTN121(28 °C)	2640	2626	2551	2606 ± 48

**Table 3:** Physicochemical properties of the nature water.

	pH	TOC (mg/L)	TP (mg/L)
W-CH	7.27	2.702	0.21
W-W	7.89	5.969	0.15
W-ZH	8.02	3.251	0.17

W-CH, W-W and W-ZH represent water samples collected from upper Chongqing, middle Wuhan and downstream Zhenjiang reaches of the Yangtze River, respectively.

**Table 4:** Concentrations (μg/L) of DBP and dry weight of mycelia at the 50 day’s cultivation of the three fungi in natural water at 15 °C (repetition times = 3).

Sample No.	First test(μg/L)	Duplicated(μg/L)	Triplicated(μg/L)	Average(μg/L)	Dry weight ofmycelia (mg)
**W-CH-Blank**	16.5	16.9	18.1	17.1 ± 0.79	1.3 ± 0.21
W-CH-PTN7	42.8	43.6	41.4	42.6 ± 1.18	21.1 ± 1.24
W-CH-PTN42	20.6	21.4	21.8	21.2 ± 0.59	4.2 ± 0.37
W-CH-PTN121	30.2	31.2	29.7	30.4 ± 0.79	8.5 ± 0.68
**W-W-Blank**	10.4	12.8	11.0	11.4 ± 1.18	1.8 ± 0.16
W-W-PTN7	17.9	18.5	19.0	18.5 ± 0.59	16.4 ± 1.87
W-W-PTN42	18.7	19.4	18.5	18.9 ± 0.60	3.6 ± 0.49
W-W-PTN121	33.2	33.8	37.1	34.8 ± 2.16	9.6 ± 0.72
**W-ZH-Blank**	11.4	13.6	12.2	12.4 ± 1.18	1.9 ± 0.21
W-ZH-PTN7	17.7	18.5	19.2	18.5 ± 0.79	15.8 ± 0.98
W-ZH-PTN42	19.0	18.7	19.4	19.0 ± 0.39	2.9 ± 0.32
W-ZH-PTN121	33.6	35.0	34.0	34.2 ± 0.79	11.6 ± 0.56

W-CH, W-W and W-ZH represent water samples respectively collected from upper Chongqing, middle Wuhan and downstream Zhenjiang reaches of the Yangtze River. The dates of weight of mycelia are measured by the dry weight of mycelium culture in 200 ml nature water.
