# Supplementary material for: Bio-Source of di-n-butyl phthalate production by filamentous fungi
Source: Sci Rep. 2016 Feb 9;6:19791. doi: 10.1038/srep19791 (PMC4746570; doi:10.1038/srep19791)
Supplement: Supplementary Information [file srep19791-s1.doc]

**SUPPLEMENTARY INFORMATION** for

Bio-Source of di-n-butyl phthalate from filamentous fungi

Cunkui Tian, Jinren Ni*, Fang Chang, Sitong Liu, Nan Xu, Weiling Sun, Yuan Xie, Yongzhao Guo, Yanrong Ma, Zhenxing Yang, Chengyuan Dang, Yuefei Huang, Zhexian Tian, Yiping Wang

E-mail address: nijinren@iee.pku.edu.cn

Tel: +86-10-62751185; fax: +86-10-62756526.

Address: College of Environmental Science and Engineering, Peking University, Beijing 100871, China

**Table of Contents**

**Supplementary Figures**

**Supplementary Fig.S1:** Chromatographs of metabolite time series obtained from HPLC for the control case and the three fungal strains cultured in artificial media at 28 °C.

The labels, 1, 2 and 3, refer to spore suspensions of 20 µL, 200 µL, and 2000 µL, respectively for the three fungi inoculated into 500 mL flasks with 200 mL of liquid medium. The experiment was repeated three times, and this figure shows the HPLC chromatograph results of an experiment repeated three times. The A, B and C, indicate this test was repeated three times. Here, the retention time is in the range from 46 to 56 minutes.The different curves on each sub-plot refer to particular days.

**Supplementary Fig.S2:** ^1^H and ^13^C NMR spectral data of DBP

**Supplementary** **Fig.S3:** Chromatographs of metabolite time series obtained from HPLC for the controls. Control 1 is the chromatograph of metabolite time series obtained from HPLC for PDA extraction, which is for the liquid medium control case; Controls 2-7 are the chromatographs of metabolite time series obtained from HPLC for phthalic acid + n-butyl alcohol, protocatechuic acid + n-butyl alcohol, protocatechuic acid, D-glucose + n-butyl alcohol, D-glucose and enzyme, which are the control cases of enzyme-mediated reaction.

**Supplementary Fig.S4:**  Chromatographs of extracellular metabolite time series obtained from HPLC for the control case and the three fungal strains cultured in artificial media at 28 °C. 200 µL spore suspensions was inoculated into 500 mL flasks with 200 mL of liquid medium. The experiment was repeated three times, and this figure shows the HPLC chromatograph results of an experiment repeated three times. The a, b and c, indicate this test was repeated three times. Here, the retention time is in the range from 46 to 56 minutes. The different curves on each sub-plot refer to particular days.

**Supplementary Fig.S5:** Chromatographs of metabolite time series obtained from HPLC for the control case and the three fungal strains cultured in artificial media at 28 °C where the background DBP concentration is varied. The spore suspension of three fungi inoculated into each of 500 mL flasks with 200 mL of liquid medium was 200 µL. This figure shows the HPLC chromatograph results of an experiment repeated three times, with the retention time selected to be in the range between 46 and 56 minutes.

**Supplementary Fig.S6:** Chromatographs of metabolite time series obtained from HPLC for the control case and the three fungal strains cultured in artificial media at a temperature of 15 °C. The spore suspension of three fungi inoculated into 500 mL flasks with 200 mL of liquid medium was 200 µL. This figure shows the HPLC chromatograph results of an experiment repeated three times, with the retention time selected to be in the range between 0 and 55 minutes.

**Supplementary Fig.S7:** Chromatographs of metabolite time series obtained from HPLC for the control case and the three fungal strains cultured in natural water at 15°C. The spore suspension of three fungi inoculated into 500 mL flasks with 200 mL of liquid medium was 200 µL. This figure shows the HPLC chromatograph results of an experiment repeated three times, with the retention time selected to be in the range between 40 and 60 minutes.

**Supplementary Tables**

**Supplementary Table S1:**  Colony-Forming Units (CFU) of three fungi inoculated in liquid media

**Supplementary Table S2：**DBP concentration results at different culture times for the three fungi, using HPLC (n=3)

**Supplementary Table S3：**Dry weight of mycelium results at different culture times for the three fungi (n=3)

A

B

C

**Supplementary figure S1 Chromatographs of metabolite time series obtained from HPLC for the control case and the three fungal strains cultured in artificial media at 28 °C.** The labels, 1, 2 and 3, refer to spore suspensions of 20 µL, 200 µL, and 2000 µL, respectively for the three fungi inoculated into 500 mL flasks with 200 mL of liquid medium. The experiment was repeated three times, and this figure shows the HPLC chromatograph results of an experiment repeated three times. The A, B and C, indicate this test was repeated three times. Here, the retention time is in the range from 46 to 56 minutes. The different curves on each sub-plot refer to particular days.


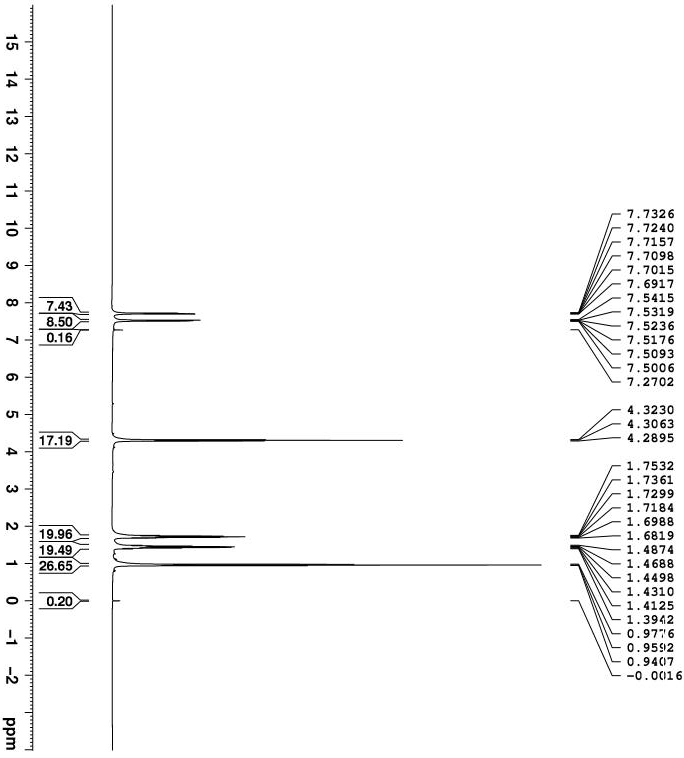


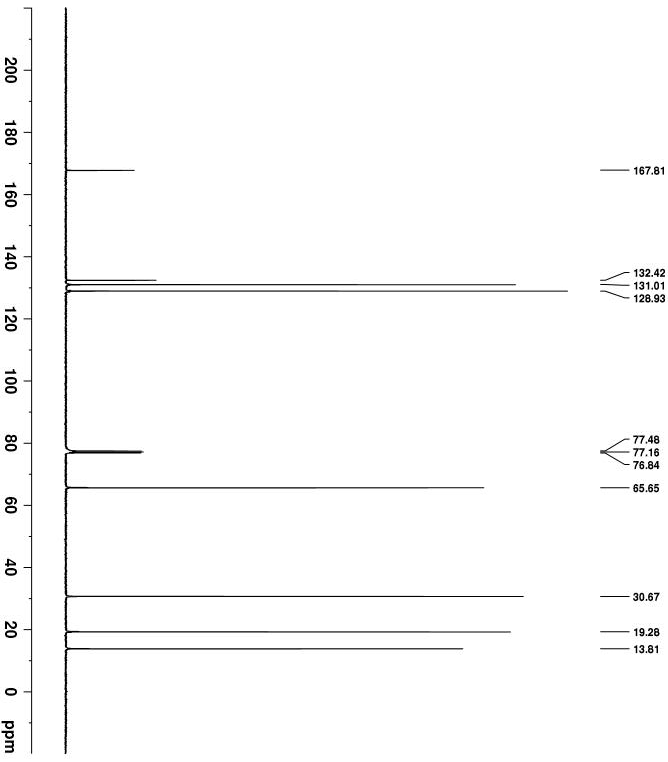


**Supplementary** **figure S2 ^1^H and ^13^C NMR spectral data of DBP** Solvent: CDCl_3_

**Supplementary** **figure S3 Chromatographs of metabolite time series obtained from HPLC for the controls.** Control 1 is the chromatograph of metabolite time series obtained from HPLC for PDA extraction, which is for the liquid medium control case; Controls 2-7 are the chromatographs of metabolite time series obtained from HPLC for phthalic acid + n-butyl alcohol, protocatechuic acid + n-butyl alcohol, protocatechuic acid, D-glucose + n-butyl alcohol, D-glucose and enzyme, which are the control cases of enzyme-mediated reaction.

**Supplementary figure S4 Chromatographs of extracellular metabolite time series obtained from HPLC for the control case and the three fungal strains cultured in artificial media at 28 °C.** 200 µL spore suspensions was inoculated into 500 mL flasks with 200 mL of liquid medium. The experiment was repeated three times, and this figure shows the HPLC chromatograph results of an experiment repeated three times. The a, b and c, indicate this test was repeated three times. Here, the retention time is in the range from 46 to 56 minutes. The different curves on each sub-plot refer to particular days.

**Supplementary figure S5 Chromatographs of metabolite time series obtained from HPLC for the control case and the three fungal strains cultured in artificial media at 28 °C where the background DBP concentration is varied.** The spore suspension of three fungi inoculated into each of 500 mL flasks with 200 mL of liquid medium was 200 µL. This figure shows the HPLC chromatograph results of an experiment repeated three times, with the retention time selected to be in the range between 46 and 56 minutes.

**Supplementary figure S6 Chromatographs of metabolite time series obtained from HPLC for the control case and the three fungal strains cultured in artificial media at a temperature of 15 °C.** The spore suspension of three fungi inoculated into 500 mL flasks with 200 mL of liquid medium was 200 µL. This figure shows the HPLC chromatograph results of an experiment repeated three times, with the retention time selected to be in the range between 0 and 55 minutes.

**Supplementary** **figure S7 Chromatographs of metabolite time series obtained from HPLC for the control case and the three fungal strains cultured in natural water at 15 °C.** The spore suspension of three fungi inoculated into 500 mL flasks with 200 mL of liquid medium was 200 µL. This figure shows the HPLC chromatograph results of an experiment repeated three times, with the retention time selected to be in the range between 40 and 60 minutes.

| **Supplementary Table S1**  **Colony-Forming Units (CFU) of three fungi inoculated in liquid media** | | | |
| --- | --- | --- | --- |
| Strains | CFU | | |
|  | 20 µL | 200 µL | 2000 µL |
| *P. lanosum* PTN121 | 2.4×10^5^ | 2.4×10^6^ | 2.4×10^7^ |
| *A.niger* PTN42 | 2.8×10^5^ | 2.8×10^6^ | 2.8×10^7^ |
| *T.asperellum* PTN7 | 1.0×10^5^ | 1.0×10^6^ | 1.0×10^7^ |
| The concentrations of spore suspension of *T.asperellum* PTN7, *A.niger* PTN42, and *P. lanosum* PTN121 are 0.5×10^7^, 1.4×10^7^, and 1.2×10^7^ cfu/mL, respectively. | | | |

| **Supplementary**  **Table S2**  **DBP concentration results at different culture times for the three fungi, using HPLC (times of repetition=3)** | | | | | | | | | | |  | |
| --- | --- | --- | --- | --- | --- | --- | --- | --- | --- | --- | --- | --- |
| *Strains* | **DBP concentration results (µg/L) at different culture times (day)** | | | | | | | | | | | |
|  | 1d | 2d | 3d | 4d | 5d | 6d | 7d | 9d | 11d | 13d | | 16d |
| *T.asperellum* PTN7-1 | 56±3 | 127±8 | 170±14 | 186±6 | 200±7 | 440±19 | 597±16 | 1499±34 | 2465±50 | 2831±79 | | 2913±70 |
| *T.asperellum* PTN7-2 | 66±2 | 211±10 | 214±31 | 231±9 | 235±12 | 820±30 | 883±28 | 1579±25 | 2612±59 | 2856±28 | | 2857±42 |
| *T.asperellum* PTN7-3 | 83±4 | 350±6 | 582±8 | 620±5 | 712±22 | 841±36 | 866±20 | 1639±41 | 2533±21 | 2841±13 | | 2895±52 |
| *A.niger* PTN42-1 | 0±0 | 372±11 | 412±5 | 630±19 | 744±5 | 913±20 | 941±9 | 1632±31 | 1863±36 | 2313±70 | | 2345±26 |
| *A.niger* PTN42-2 | 0±0 | 375±9 | 518±13 | 658±16 | 753±21 | 1038±25 | 1064±22 | 1789±34 | 2024±40 | 2540±27 | | 2504±51 |
| *A.niger* PTN42-3 | 302±13 | 599±16 | 596±30 | 639±18 | 824±24 | 1354±31 | 1413±35 | 1840±36 | 1981±39 | 2489±50 | | 2544±51 |
| *P. lanosum* PTN121-1 | 0±0 | 198±5 | 659±22 | 850±19 | 891±10 | 959±18 | 1138±26 | 1697±33 | 2235±39 | 2536±28 | | 2547±14 |
| *P. lanosum* PTN121-2 | 0±0 | 307±15 | 711±12 | 893±25 | 1059±19 | 1169±17 | 1203±38 | 1818±51 | 2384±44 | 2570±36 | | 2606±48 |
| *P. lanosum*PTN121-3 | 0±0 | 658±11 | 875±20 | 1057±10 | 1203±28 | 1501±15 | 1498±31 | 1890±49 | 2446±51 | 2581±48 | | 2565±16 |

The numbers (-1, -2 and -3) after the name of the fungi indicate the amount of spore suspensions (20 µL, 200 µL and 2000 µL) add to 200 mL of liquid medium.

| **Supplementary Table S3**  **Dry weight of mycelium results at different culture times for the three fungi (times of repetition=3)** | | | | | | | | | | | |
| --- | --- | --- | --- | --- | --- | --- | --- | --- | --- | --- | --- |
| Strains | **Dry weight of mycelium results (mg) at different culture times (day)** | | | | | | | | | | |
|  | 1d | 2d | 3d | 4d | 5d | 6d | 7d | 9d | 11d | 13d | 16d |
| *T.asperellum* PTN7-1 | 18±1 | 22±2 | 29±3 | 33±3 | 44±2 | 131±3 | 157±5 | 192±3 | 234±16 | 282±9 | 266±10 |
| *T.asperellum* PTN7-2 | 19±2 | 23±3 | 31±3 | 32±2 | 52±3 | 167±4 | 209±8 | 244±6 | 338±12 | 313±7 | 283±11 |
| *T.asperellum* PTN7-3 | 23±2 | 23±2 | 108±6 | 114±5 | 111±5 | 170±9 | 238±6 | 350±8 | 378±10 | 354±6 | 348±7 |
| *A.niger* PTN42-1 | 14±2 | 16±2 | 17±2 | 20±2 | 31±4 | 32±2 | 36±2 | 51±3 | 43±3 | 41±3 | 8±3 |
| *A.niger* PTN42-2 | 17±3 | 25±3 | 26±2 | 32±3 | 31±2 | 38±4 | 42±3 | 132±4 | 154±4 | 79±5 | 74±4 |
| *A.niger* PTN42-3 | 7±2 | 12±2 | 15±3 | 34±2 | 43±3 | 112±6 | 128±7 | 164±3 | 209±7 | 163±3 | 87±7 |
| *P. lanosum* PTN121-1 | 16±2 | 20±2 | 30±3 | 51±4 | 68±7 | 73±5 | 78±5 | 86±6 | 125±6 | 184±14 | 65±6 |
| *P. lanosum* PTN121-2 | 19±3 | 34±4 | 40±2 | 66±6 | 77±3 | 101±3 | 103±4 | 112±8 | 147±7 | 227±7 | 114±3 |
| *P. lanosum* PTN121-3 | 23±2 | 42±3 | 52±2 | 60±5 | 83±2 | 112±6 | 167±4 | 188±5 | 203±6 | 249±8 | 171±10 |

The numbers (-1, -2 and -3) after the name of the fungi indicate the amount of spore suspensions (20 µL, 200 µL and 2000 µL) add to 200 mL of liquid medium. The dates in the table are measured by the dry weight of mycelium in 20ml fermentation broth.
